# Immune Checkpoint Inhibition as Primary Adjuvant Therapy for an *IDH1*-Mutant Anaplastic Astrocytoma in a Patient with CMMRD: A Case Report—Usage of Immune Checkpoint Inhibition in CMMRD

**DOI:** 10.3390/curroncol28010074

**Published:** 2021-02-01

**Authors:** Rebekah Rittberg, Craig Harlos, Heidi Rothenmund, Anirban Das, Uri Tabori, Namita Sinha, Harminder Singh, Bernie Chodirker, Christina A. Kim

**Affiliations:** 1 Section of Hematology/Oncology, Department of Internal Medicine, Rady Faculty of Health Sciences, University of Manitoba, Winnipeg, MB R3E 0V9, Canada; charlos@cancercare.mb.ca (C.H.); ckim3@cancercare.mb.ca (C.A.K.); 2 Department of Biochemistry and Medical Genetics, Max Rady College of Medicine, University of Manitoba, Winnipeg, MB R3E 0J9, Canada; hrothenmund@hsc.mb.ca (H.R.); BChodirker@hsc.mb.ca (B.C.); 3 Division of Hematology/Oncology, The Hospital for Sick Children, Department of Pediatrics, University of Toronto, Toronto, ON M5G 1X8, Canada; anirban.das@sickkids.ca (A.D.); uri.tabori@sickkids.ca (U.T.); 4 Department of Pathology, Rady Faculty of Health Sciences, University of Manitoba, Winnipeg, MB R3E 3P5, Canada; nsinha@sharedhealthmb.ca; 5 Section of Gastroenterology, Department of Internal Medicine, Rady Faculty of Health Sciences, University of Manitoba, Winnipeg, MB R3A 1R9, Canada; Harminder.singh@umanitoba.ca; 6 Research Institute in Oncology and Hematology, CancerCare Manitoba, Winnipeg, MB R3E 0V9, Canada

**Keywords:** constitutional mismatch repair deficiency, CMMRD, immunotherapy, adjuvant therapy, checkpoint inhibitors, screening, surveillance, tumor mutational burden

## Abstract

Constitutional mismatch repair deficiency (CMMRD) is a rare autosomal recessive hereditary cancer syndrome due to biallelic germline mutation involving one of the four DNA mismatch repair genes. Here we present a case of a young female with CMMRD, homozygous for the c.2002A>G mutation in the *PMS2* gene. She developed an early stage adenocarcinoma of the colon at the age of 14. Surveillance MRI of the brain at age 18 resulted in the detection of an asymptomatic brain cancer. On resection, this was diagnosed as an anaplastic astrocytoma. Due to emerging literature suggesting benefit of immunotherapy in this patient population, she was treated with adjuvant dual immune checkpoint inhibition, avoiding radiation. The patient remains stable with no evidence of progression 20 months after resection. The patient’s clinical course, as well as the rational for considering adjuvant immunotherapy in patients with CMMRD are discussed in this report.

## 1. Introduction

Constitutional mismatch repair deficiency (CMMRD), first described in 1999 [1], is an autosomal recessive syndrome due to biallelic germline mutation involving one of the four DNA mismatch repair (MMR) genes (*MLH1*, *MSH2*, *MSH6*, or *PMS2*) [2,3]. The MMR genes play a critical role in correcting single base pair mismatch and short insertions/deletions, ensuring genome stability [4,5]. CMMRD is associated with high morbidity due to the onset of malignancies during childhood or early adulthood [6,7]. The most common CMMRD-related malignancies are hematologic and gastrointestinal cancers; however, other cancer types including endometrial, ovarian, urinary tract, and sarcomas have been described [7,8].

The first malignancy in CMMRD is usually diagnosed within the first decade of life [7]. Brain tumors are common, occurring in 55% of patients, with high grade glioma (HGG) being the predominant pathology, presenting at a mean age of diagnosis of nine years [9]. CMMRD patients may also have non-malignant manifestations, including café-au-lait macules, hypopigmentation, and immunoglobulin deficiencies [2,3,10]. Presentation varies based on the impacted MMR gene. Onset of first malignancy occurs later in patients with biallelic germline mutation of *PMS2*, with the mean age of first malignancy of 10 years, compared to 7.5 and 8 years for patients with biallelic loss of *MSH6* and *MLH1*/*MSH2,* respectively [9,11]. Biallelic *PMS2* mutation is also associated with a greater incidence of brain tumors, colorectal cancer (CRC), and endometrial carcinomas compared to biallelic mutations in *MLH1*, *MSH2,* and *MSH6* which have a proportionally higher rate of hematologic malignancies [7]. Cancer surveillance is a critical component of CMMRD management and a number of surveillance guidelines exist (Table 1) [6,11,12,13]. Variation between these protocols may be due to differences in the CMMRD population assessed when guidelines were published [6,11,12,13]. Whole body MRI (WBMRI) has emerged as a potentially useful screening tool [6,12] and patient series suggest that WBMRI may improve early detection [14] and survival [15].

As CMMRD is rare, with less than 200 cases reported in the literature, surveillance guidelines and optimal cancer treatment options are still evolving [6,9,12]. CMMRD is likely under recognized, and is likely more prevalent in endogamous population [16], highlighting the importance of maintaining a high clinical suspicion. Here we describe a case of a young female with CMMRD diagnosed with colon cancer and an *IDH*-mutant anaplastic astrocytoma (AA). To our knowledge, this is the first published case of a CMMRD associated IDH-mutant AA treated with adjuvant nivolumab and ipilimumab after resection, without adjuvant radiation.

## 2. Case Description

Here we present a case of a 19-year-old Inuit female who comes from a family known to have CMMRD following the diagnosis of gastric cancer in a sibling at age 11. Our patient underwent molecular testing at age 10, which confirmed she was homozygous for the c.2002A>G mutation in the *PMS2* gene. This variant is classified as likely deleterious and has been described as a founder mutation in the Inuit population of northern Quebec [17]. She was included as part of that series.

The patient subsequently began surveillance gastroscopies and colonoscopies at age 11. At age 14, she was diagnosed with a pT2N0 moderately differentiated adenocarcinoma of the colon, treated with total colectomy. MMR immunohistochemistry showed loss of expression of *PMS2* in both neoplastic epithelial and normal mesenchymal elements, a finding specific for CMMRD [3]. Since 2018, she has been followed in the Manitoba Hereditary Gastrointestinal Cancer Clinic, where surveillance recommendations were followed based on current guidelines [6,11,12,13].

**Figure 1:**
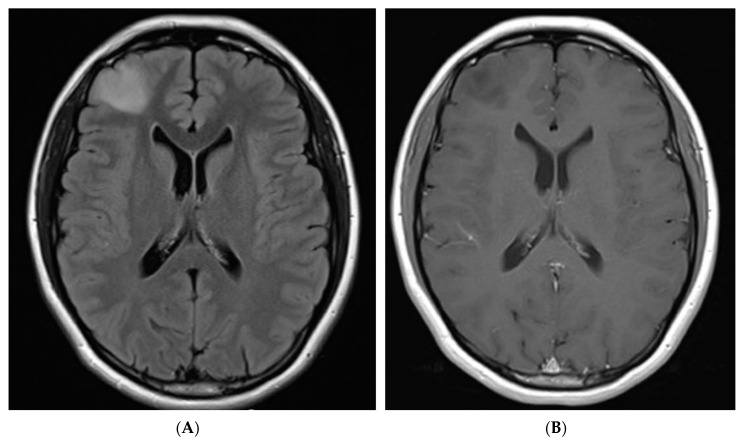

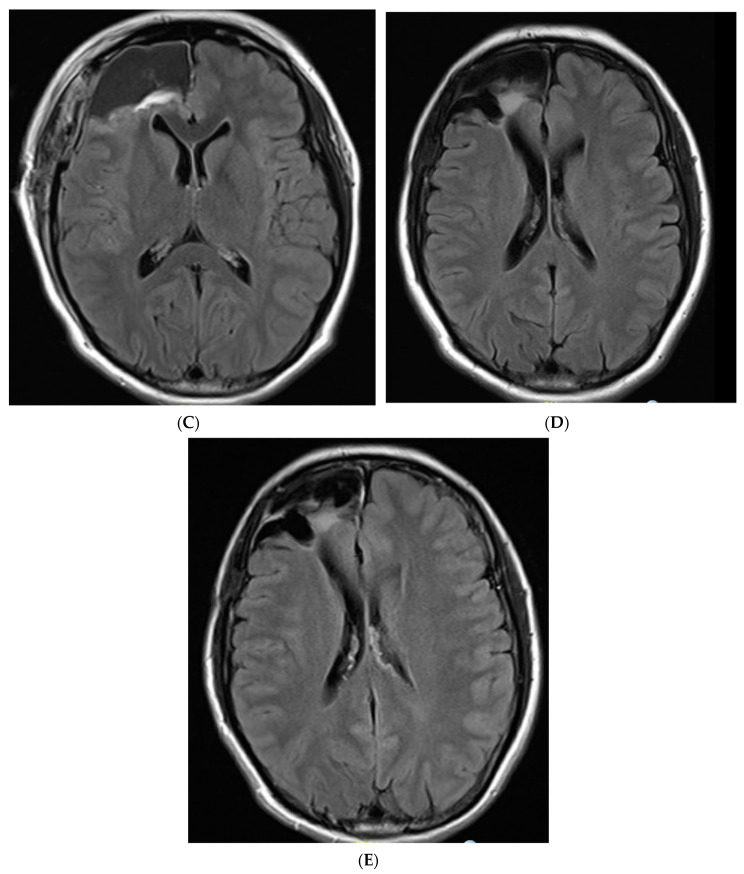
FLAIR sequence axial MRI brain images. (**A**) 2.6 × 2.0 cm lesion in right frontal lobe (pre-operative). (**B**) T1 post contrast pre-operative image of right frontal lobe lesion (pre-operative) (**C**) Hyperintense blood products in resection bed (post-operative day 1), (**D**) Focal area of increased T2/FLAIR signal deep to resection cavity (prior to starting ICI, 9 months after resection). (**E**) T2/FLAIR hyperintense tissue changes in right frontal lobe appear stable (18 months post resection).

**Figure 2:**
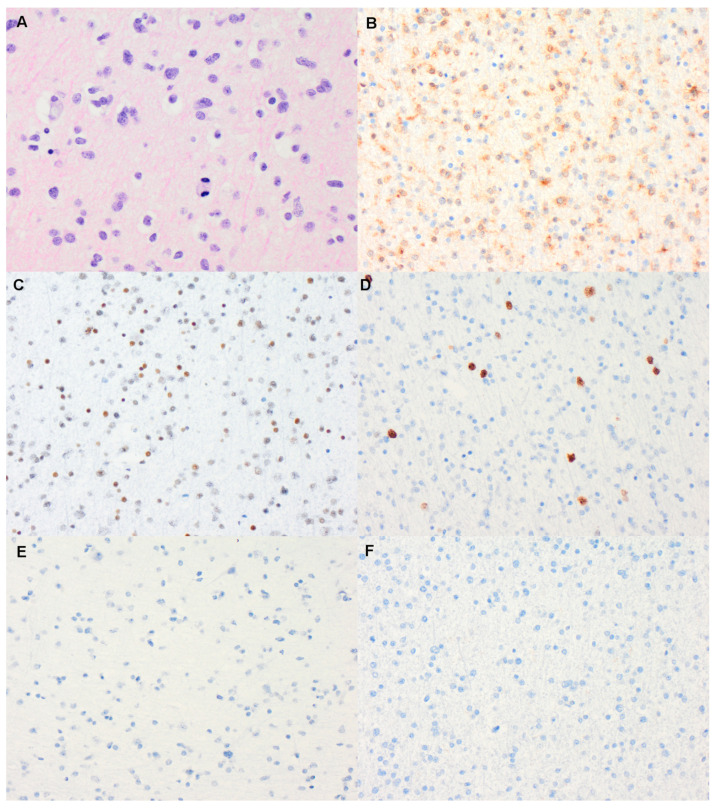
(**A**) Hematoxylin and eosin stain stained section showing an infiltrating anaplastic astrocytoma, composed of mitotically active astroglial cells with enlarged, hyperchromatic, irregular nuclei (×400 magnification). On immunohistochemistry, the tumor cells are positive for mutant *IDH1* R132H (**B**); show loss of ATRX expression in tumor nuclei (**C**); a small subset of tumor nuclei expressing p53 (**D**); complete loss of *PMS2* in both normal brain tissue and neoplastic astroglial cells (**E**); and absence of PDL1 expression (**F**) (all images at ×200 magnification).

On first surveillance brain MRI, at age 18, she was found to have a 2.6 cm mass-like area of FLAIR hyperintensity in the right frontal lobe (Figure 1). She underwent a craniotomy and resection of tumor. Pathology demonstrated a HGG with frequent mitosis but no definite endothelial proliferation and/or necrosis and she was diagnosed with AA, *IDH1 R132H* mutant, WHO grade 3, with immunohistochemistry results suggestive of biallelic *PMS2* mutation (Figure 2). Immunohistochemistry was negative for BRAF V600E mutation and PD1/PDL1 expression. Whole exome sequencing identified a tumor mutational burden (TMB) of 8 mutations/Mb [18]. In addition to the *IDH1 R132H* mutation, a pathogenic variant in TP53 was detected (c.817C>T; p.Arg273Cys).

An MRI brain 12 weeks after surgical resection demonstrated a slight increase of the non-enhancing region deep to the resection cavity, suggestive of possible residual disease. There was new increased signal intensity on FLAIR along the superior and deep margin of the resection cavity. Because of the rarity of this condition, her case was discussed in a multidisciplinary fashion with experts across Canada. Emerging literature suggests that high-grade brain malignancies in children with CMMRD can demonstrate objective responses and prolonged survival following immune checkpoint inhibition (ICI) [19,20]. As such, she was offered adjuvant nivolumab and ipilimumab; however, treatment initiation was delayed for nine months due to pregnancy. Uninfused MRI brain was completed every 2–3 months during pregnancy, with a non-contrast MRI brain in the third trimester of pregnancy demonstrating slight increase in bulk of the FLAIR changes. These findings were stable on the post pregnancy MRI. Despite treatment delay, after delivery, adjuvant nivolumab (3 mg/kg) and ipilimumab (1 mg/kg) was administered every three weeks for four cycles, followed by maintenance nivolumab. After multidisciplinary discussion, as the possible residual disease had remained stable, the consensus was to forgo radiotherapy.

After cycle 1 of nivolumab and ipilimumab, she developed grade 1, clinically asymptomatic hyperthyroidism (TSH < 0.015, free T3 44, free T4 > 100). She was assessed by endocrinology, and the differential diagnosis was postpartum thyroiditis versus thyroid dysfunction secondary to immunotherapy (thyroperoxidase antibody was within normal limits at 18 IU/mL). Immunotherapy continued with close monitoring of clinical symptoms and bloodwork with gradual improvement in her thyroid function tests. After cycle 4, she developed headache, decreased appetite, and fatigue. Bloodwork confirmed grade 2 hypothyroidism (TSH > 100, free T3 < 1.0, free T4 1.0) and grade 2 adrenal insufficiency (random cortisol 38 nmol/L). MRI showed immune-related hypophysitis. Maintenance nivolumab was held and she was treated with levothyroxine, as well as a tapering course of prednisone followed by replacement hydrocortisone. Hospitalization was not required. No other immune-related adverse events were experienced. A brain MRI demonstrated stable post-treatment changes prior to the re-initiation of maintenance nivolumab. Her MRI continues to show stability with no evidence of progression 20 months post-resection and 8 months post-hypophysitis.

## 3. Discussion

Gliomas are classified and graded based on histologic and molecular features. Grade 3 (AA) and 4 (glioblastoma) gliomas are classified as HGG. Standard treatment is dependent on these characteristics, with AA being treated with concurrent radiation and temozolomide followed by adjuvant temozolomide [21]. Pediatric HGG have a median overall survival of only 7–15 months despite multimodal treatment using surgical resection, radiation, and chemotherapy [22,23,24]. CMMRD-associated HGG are particularly aggressive cancers with a very poor prognosis. They are resistant to alkylating antineoplastic agents, including temozolomide which is frequently used in the treatment of HGG [25]. Further, continuous accumulation of mutations related to the germline defect make them rapidly refractory to conventional treatments. Specifically, *IDH1* mutant MMR-deficient HGG have been documented to have a median OS of 15 months with conventional chemo-irradiation based approaches [26].

A recent report from an international consortium demonstrated that early detection of cancers using systematic surveillance can significantly improve survival in patients with CMMRD, including those with malignant brain tumors [15]. As CMMRD-related HGGs are aggressive and rapidly fatal malignancies, lead-time bias is unlikely to play a role in the actual reduction of mortality and improvement in survival following surveillance in these cancers. It is plausible that in the setting of background DNA mismatch repair deficiency, early detection and adequate surgical excision can prevent accumulation of additional deleterious mutations that develop in these tumors with time and prevent these cancers from developing a more aggressive phenotype [18,27]. However further prospective studies are required to delineate whether surveillance protocols consistently result in improved all-cause mortality in CMMRD. In our case, surveillance led to the early detection and treatment of both her asymptomatic gastrointestinal and CNS malignancies. Whether this will result in improved long term outcomes for our patient is not known, but highlights the importance of a multidisciplinary team, which can facilitate regular surveillance and implement appropriate treatment options.

In many areas of oncology, ICI have provided promise and dramatically altered the landscape in cancer treatments and outcomes, predominantly in tumors with high mutation burden, microsatellite-instability and/or an immune microenvironment [28,29]; however, the use of ICI in adult gliomas has not demonstrated satisfactory responses [30,31]. This is related to multiple factors, including lower mutation burden, acquiring mutations later in tumorigenesis, relative paucity of indels, poor neoantigen quality, high sub-clonality, and an immunosuppressed microenvironment in adult HGG [30,32]. In contrast, case reports in children with HGG and CMMRD have demonstrated favorable responses to ICI. AlHarbi et al. published a case of a five-year-old with CMMRD with an incurable HGG who experienced a durable treatment response of at least 10 months with nivolumab [33]. Bouffet et al. published a case series of two siblings with recurrent multifocal glioblastoma treated with ICI, with disease stability for 11 and 30 months post-recurrence [19,34]. The international consortium has recently reported on the impressive responses and survival of high-grade brain tumors in CMMRD using ICI [15]. These responses are possibly related to the early onset, and continuous accumulation [18] of high numbers of single nucleotide variants, total and microsatellite indels [35], which drive a robust immune response in this young cohort of patients with an underlying germline defect [29].

The KEYNOTE 158 study evaluated pembrolizumab in tumors with high mutational burden (≥10 mutations/Mb) and found a 29% response rate, resulting in Food & Drug Administration (FDA) approval of pembrolizumab for this indication in June 2020 [36]. Because our patient had a TMB of eight mutations/Mb, she would not have met this FDA indication. However, the cutoff for high TMB varies across tumor types [37,38]. The index tumor at the time of biopsy harbored eight mutations/Mb, which is much higher compared to the median mutation burden for pediatric high-grade brain tumors and gliomas (<1 to 1.8 mutation/Mb) [39]. Though the current FDA approval for the use of Pembrolizumab proposes a TMB > 10 mutations/Mb, there is increasing evidence that responses to ICI cannot be predicted by single biomarker cut-off [40]. Lombardi et al. completed a pilot study evaluating pembrolizumab in 13 adult patients with recurrent HGG with complete or partial loss of MMR protein expression showed a disease control rate of 31% and response to therapy did not correlate with degree of TMB [41]. However, pediatric HGG differ from adult HGG and an ongoing clinical trial is therefore prospectively evaluating the efficacy of ICI in pediatric tumors with mutation burden exceeding five mutations/Mb among other indications (NCT02992964) [42].

To our knowledge, we present the first case of a patient with a CMMRD associated HGG, detected at an asymptomatic stage using systematic surveillance, and treated using adjuvant nivolumab and ipilimumab following surgical resection. She is alive 20 months following her diagnosis. The prolonged survival following ICI is remarkable for a rapidly fatal and aggressive cancer. The additional opportunity to omit radiotherapy, thereby eliminating its late sequelae, may offer a new paradigm for management of these patients in the future.

## Figures and Tables

**Table 1:** European Consortium, Canadian and US Multi-society Task Force on Colorectal Cancer Constitutional mismatch repair deficiency (CMMRD) surveillance guidelines.

Malignancies	European Consortium [11]	Canadian Surveillance Protocol [6,12]	US Multi-Society Task Force on Colorectal Cancer [13]
Brain tumors	MRI brain every 6 to 12 months, starting at the age of 2	MRI brain at diagnosis then every 6 months	MRI brain every 6 months, starting at the age of 2
Gastrointestinal tumors	Upper: annual video capsule endoscopy and gastroscopy, starting at the age of 10	Upper: annual video capsule endoscopy and gastroscopy, starting between 4 to 6 years	Upper: annual video capsule endoscopy and gastroscopy, starting at the age of 8
	Lower: annual colonoscopy, starting at the age of 8	Lower: annual colonoscopy, starting between 4 to 6 years	Lower: annual colonoscopy, starting at the age of 6
Hematologic malignancies	Annual clinical examination and CBC every 6 months, starting at the age of 1	Abdominal ultrasound every 6 months, starting at the age of 1	CBC every 6 months, starting at the age of 1
	Optional: abdominal ultrasound every 6 months, starting at the age of 1	CBC every 6 months, starting at the age of 1	
Genitourinary tumors	Annual urine cytology and urine dipstick, starting at the age of 20	Annual urine cytology and urine dipstick, starting at the age of 20	Annual urinalysis, starting at the age of 10
Gynecologic tumors	Annual gynecologic examination, transvaginal ultrasounds, and endometrial biopsy, starting at the age of 20	Annual gynecologic examination, transvaginal ultrasounds, and endometrial biopsy, starting at the age of 20	Annual gynecologic examination, transvaginal ultrasounds, and endometrial biopsy, starting at the age of 20

Abbreviations: MRI = magnetic resonance imaging, CBC = complete blood count.

## Data Availability

No new data were created or analyzed in this study. Data sharing is not applicable to this article.
